# Predictive value of left atrial volumes assessed using real-time three-dimensional echocardiography for pulmonary hypertension in dogs with myxomatous mitral valve disease

**DOI:** 10.3389/fvets.2024.1441839

**Published:** 2024-08-16

**Authors:** In-Sun Woo, Jung-Hyun Kim

**Affiliations:** Department of Veterinary Internal Medicine, College of Veterinary Medicine, Konkuk University, Seoul, Republic of Korea

**Keywords:** dog, left atrial volume, myxomatous mitral valve disease, pulmonary hypertension, pulmonary wedge pressure, three-dimensional echocardiography

## Abstract

**Introduction:**

Left atrial volume (LAV) obtained using real-time three-dimensional echocardiography (RT3DE) is an independent predictor of post-capillary pulmonary hypertension (PH) in humans; however, no studies have investigated LAV obtained using RT3DE as a predictor of post-capillary PH in dogs with myxomatous mitral valve disease (MMVD). Therefore, we aimed to evaluate the clinical applicability of LAV obtained using RT3DE compared to that obtained using two-dimensional echocardiography (2DE) in dogs with MMVD, with or without PH.

**Methods:**

Medical records and echocardiographic images of 237 privately owned dogs with naturally occurring MMVD with or without PH were retrospectively reviewed. A total of 49 privately owned dogs with naturally occurring MMVD, with or without PH, were finally included (35 MMVD without PH, 14 MMVD with PH). The LAV and left ventricular volumes were obtained using 2DE and RT3DE. Echocardiographic parameters were analyzed to identify independent predictors of post-capillary PH.

**Results:**

We found that the left atrial and left ventricular volumes obtained using 2DE and RT3DE indexed to body weight and several 2DE-derived variables were univariately associated with post-capillary PH. Furthermore, multivariable logistic regression analysis revealed that the RT3DE minimum LAV indexed to body weight (LAVi min) was the only significant independent predictor of post-capillary PH (odds ratio, 12.86; 95% confidence interval [CI], 2.40–68.99; *p* = 0.003), with the highest area under the curve value of 0.86 (95% CI, 0.75–0.96; *p* < 0.001).

**Discussion:**

In conclusion, LAV indexed to body weight obtained using 2DE and RT3DE, can be a useful predictor of post-capillary PH in dogs with MMVD. In particular, the RT3DE LAVi min was observed to be the strongest predictor of post-capillary PH.

## 1 Introduction

Myxomatous mitral valve disease (MMVD) is the most common cardiovascular disease in dogs and can result in severe or fatal complications, such as pulmonary hypertension (PH) and congestive heart failure (1). PH is characterized by increased pulmonary arterial pressure and has a prevalence ranging from 14 to 48% in dogs with MMVD (2). PH is hypothesized to result from chronically elevated diastolic filling pressures of the left heart (3). PH can be life-threatening and impacts diagnostic and therapeutic decisions in dogs with MMVD (1). Therefore, a precise and prompt diagnosis of PH is essential for the clinical management of dogs with MMVD.

Cardiac catheterization is the gold standard for PH diagnosis in humans. However, this method is invasive and requires general anesthesia; therefore, it is rarely performed in dogs (4). In veterinary medicine, PH is generally diagnosed by estimating pulmonary arterial pressure using Doppler echocardiography, constituting a non-invasive alternative to cardiac catheterization. Various echocardiographic variables, including the modified Bernoulli equation and tricuspid regurgitation (TR) velocity, have been investigated for PH diagnosis (4).

Among these echocardiographic variables, left atrial volume (LAV) is a prominent marker of left ventricular (LV) filling pressure and is closely related to pulmonary arterial wedge pressure (5). The LAV can be assessed using two-dimensional echocardiography (2DE) or real-time three-dimensional echocardiography (RT3DE). However, 2DE systemically underestimates LAV owing to geometric assumptions regarding the left atrial (LA) shape and foreshortening of the LA cavity (6). RT3DE-derived LAVs have been proven more accurate and reproducible than 2DE values owing to the lack of geometric assumptions and image foreshortening (7). Therefore, RT3DE has been reported to be superior to 2DE for estimating LAV in human and veterinary medicine (8, 9). However, RT3DE has several limitations in veterinary medicine, including high equipment costs, uncontrolled patient movements and respiration causing stitch artifact, and time-consuming postprocessing analysis (10).

Body size is a major factor affecting LA size; therefore, indexing LAV to a body size variable, such as body surface area (BSA) or body weight (BW), is essential for accurate comparisons (11).

Previous studies have reported LA enlargement to be a robust predictor of cardiac events, including atrial fibrillation, heart failure, and post-capillary PH, in humans (6, 12, 13). In veterinary medicine, LAV obtained using 2DE and RT3DE showed significant predictive value in dogs with MMVD (14, 15). However, to the best of our knowledge, no studies have investigated the predictive value of LAV measurements obtained using RT3DE for post-capillary PH in dogs with MMVD.

Therefore, we aimed to evaluate the clinical applicability of BW-indexed LAV measurements obtained using RT3DE compared with those obtained using 2DE in dogs with MMVD, with or without PH. We hypothesized that LA dilatation measured using RT3DE could be superior to that measured using 2DE for predicting post-capillary PH in dogs with MMVD.

## 2 Materials and methods

### 2.1 Study population

This retrospective study was approved by the Institutional Animal Care and Use Committee of Konkuk University, Republic of Korea (approval number: KU23186), which waived the requirement for obtaining informed consent. We reviewed the medical records of dogs with MMVD admitted to the veterinary medical teaching hospital at Konkuk University between February 2018 and January 2023.

**Figure 1:**
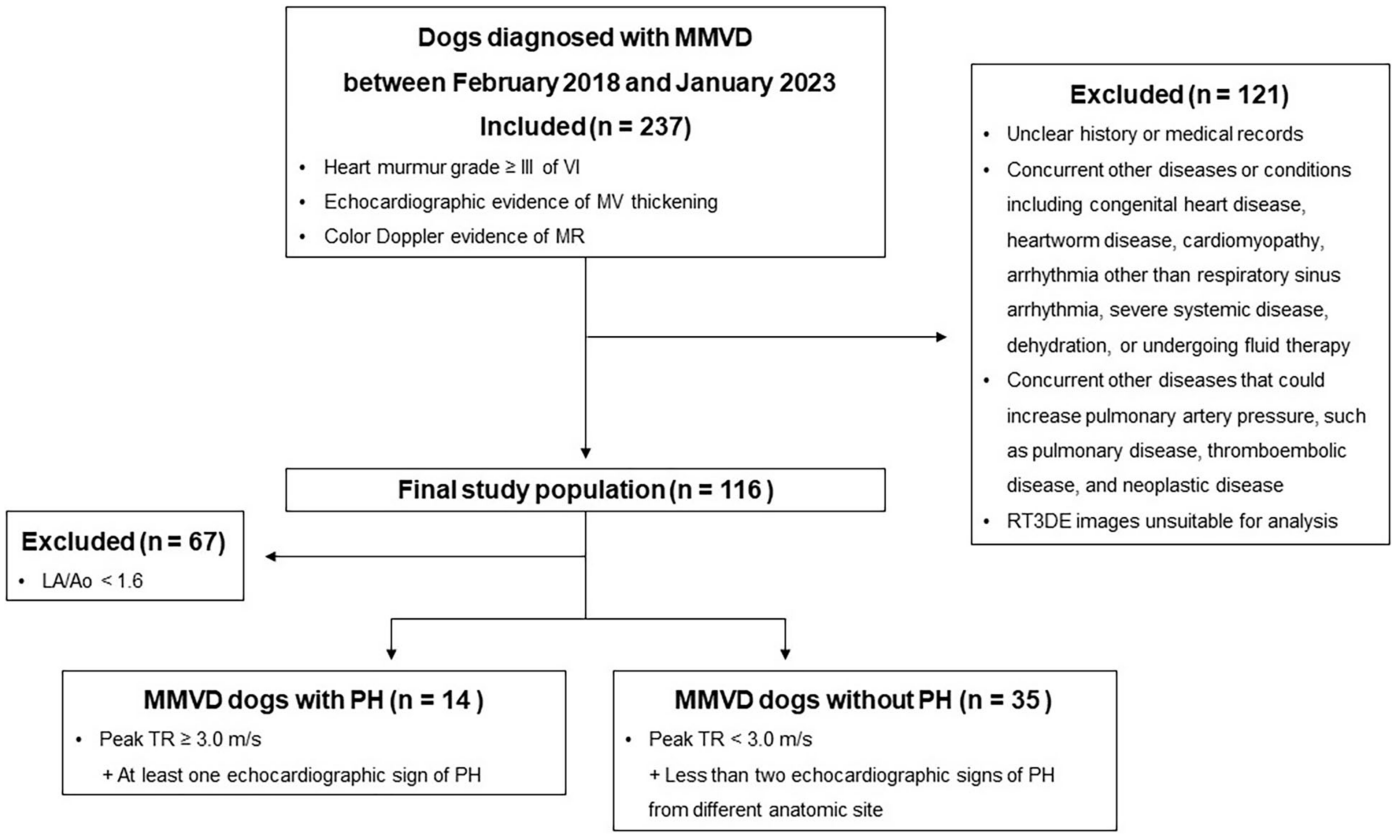
Study flowchart for inclusion and exclusion of dogs with MMVD. MMVD, Myxomatous mitral valve disease; MR, Mitral valve regurgitation; MV, Mitral valve; PH, Pulmonary hypertension; RT3DE, Real-time three-dimensional echocardiography; and TR, Tricuspid regurgitation.

The inclusion and exclusion criteria are presented in Figure 1. Assessment of pulmonary or bronchial disease was based on clinical signs and thoracic radiography. Dogs with MMVD with tricuspid regurgitation (TR; maximal velocity of ≥3.0 m/s) and other echocardiographic findings suggestive of PH were classified as dogs with MMVD and PH (at least an intermediate probability of PH) (4). Other echocardiographic findings included flattening of the interventricular septum (particularly during systole), right ventricular hypertrophy (wall thickening, chamber dilatation, or both), main pulmonary artery to aorta ratio > 1.0, peak early diastolic pulmonary regurgitation velocity (> 2.5 m/s), right ventricular outflow tract acceleration time < 52–58 ms, pulmonary artery acceleration time-to-ejection time ratio < 0.3, or systolic notching of the right ventricular outflow tract on Doppler echocardiographic profile (4).

### 2.2 Study groups

Dogs with MMVD were divided into two groups (stages B2 and C/D) based on severity, following the American College of Veterinary Internal Medicine consensus (16). Stage B2 was defined as asymptomatic dogs with more advanced MR, which can cause LA and LV enlargement. LA and LV enlargement were determined based on the following criteria: an echocardiographic LA diameter to aortic diameter ratio (LA/Ao) in early diastole ≥1.6, an LV end-diastolic internal diameter normalized for BW (LVIDDn) ≥1.7, and a radiographic vertebral heart score > 10.5. Additionally, the dogs were divided based on the presence or absence of PH.

### 2.3 Two-dimensional echocardiography and Doppler examinations

#### 2.3.1 Equipment and procedures

A single experienced veterinarian (JH-K) performed the conventional and Doppler examinations using an ultrasound unit^1^ equipped with a 3.0–8.0 MHz phased-array transducer. A conventional echocardiography protocol was conducted, as previously described (17). This involved transthoracic echocardiography, including two-dimensional imaging, M-mode, spectral, and color-flow Doppler, in addition to continuous electrocardiogram monitoring. The tissue Doppler imaging was used to assess the velocity, strain, and strain rate of the interventricular septum. All dogs were gently restrained in the right and left lateral recumbency and examined without sedation. Echocardiographic data were recorded digitally for offline analysis.

#### 2.3.2 Left heart volume and ejection fraction assessment by 2DE

**Figure 2:**
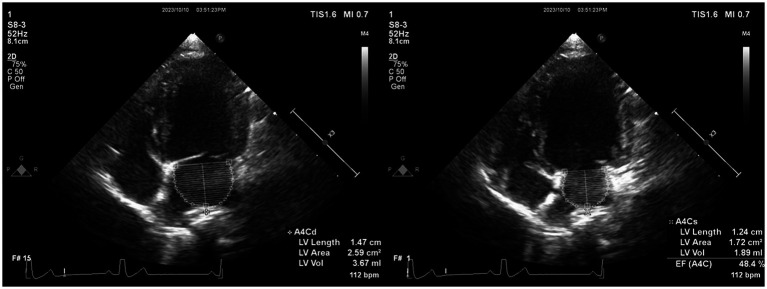
Representative image of LAV obtained using 2DE. We employed the monoplane Simpson’s method of disks to determine maximum and minimum LAVs at end-systole (just before mitral valve opening) and end-diastole (the frame after mitral valve closure) in both echocardiographic views. The inner margin of the LA was manually traced, starting from the septal mitral annulus (initial hinge point), following the contour of the LA roof, and ending at the lateral mitral annulus (secondary hinge point), excluding the pulmonary vein ostia and LA appendage. To establish the boundary of the LA, a straight line was drawn from the initial hinge point to the secondary hinge point across the mitral valve annulus. Finally, the height of the stacked disks was selected, perpendicular to the midpoint of the mitral valve annulus bisecting the LA. 2DE, Two-dimensional echocardiography; bpm, Beats per minute; EF, Ejection fraction; LA, Left atrium; and LAV, Left atrial volume.

**Figure 3:**
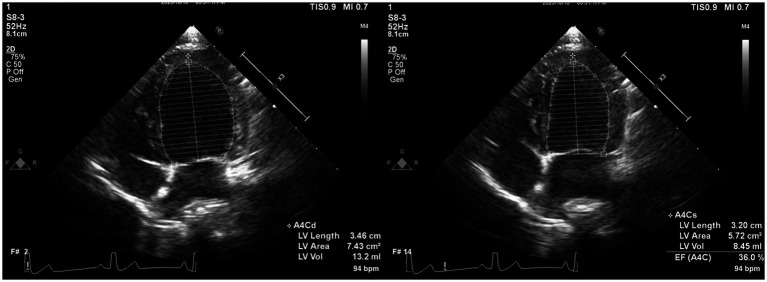
Representative image of LV volume obtained using 2DE. We employed Monoplane Simpson’s method of disks to measure LV volume from the left apical four-chamber view. The endocardial border was traced manually during diastole and systole. When the papillary muscles and trabeculae were not traced, they were manually included in the LV volume calculation. 2DE, Two-dimensional echocardiography; bpm, Beats per minute; EF, Ejection fraction; LV, Left ventricular.

Offline analyses were performed by one investigator (I-SW) using the system software.^2^ The investigator was blinded to the dogs’ statuses during the offline analysis. The monoplane Simpson’s method of disks (SMOD) was used to assess the LAV (Figure 2) (18), and monoplane SMOD LV measurements were obtained using the left apical four-chamber view (Figure 3) (11, 19), as previously described. Maximum LAV (i.e., end-systolic) assessment was performed using the frame immediately before mitral valve opening, and minimum LAV (i.e., end-diastolic) was obtained using the frame contiguous to mitral valve closure (14, 18). The LA endocardial border, excluding the LA appendage and the confluences of pulmonary veins, was traced with a straight line connecting the septal and lateral mitral leaflet base attachment points to the annulus as the superior border of the outlined area. All LAV and LV volumes obtained using 2DE were indexed to body weight (BW) (14, 18). The volumes and ejection fraction (EF) were calculated automatically using the system software (see text footnote 2).

### 2.4 Real-time three-dimensional echocardiography

#### 2.4.1 Equipment and procedures

The same experienced veterinarian (JH-K) performed the RT3DE examinations using the same ultrasound unit (see text footnote 1) used for standard echocardiography, with concomitant electrocardiogram registration. The images obtained using RT3DE were created using a matrix transducer with a frequency range of 2.0–7.0 MHz, depending on the dog size, from the apical view. The datasets were acquired using multibeat full-volume acquisition to ensure the inclusion of the entire LAV within the pyramidal scan volume, with a relatively higher volume rate. Four pyramidal-shaped sub-volumes from each complete cardiac cycle were used to produce a larger pyramidal volume and a full dataset. The transducer was adjusted for optimal acquisition of apical four-chamber and two-chamber views of the left atrium and ventricle.

#### 2.4.2 Left heart volume and ejection fraction assessment by RT3DE

**Figure 4:**
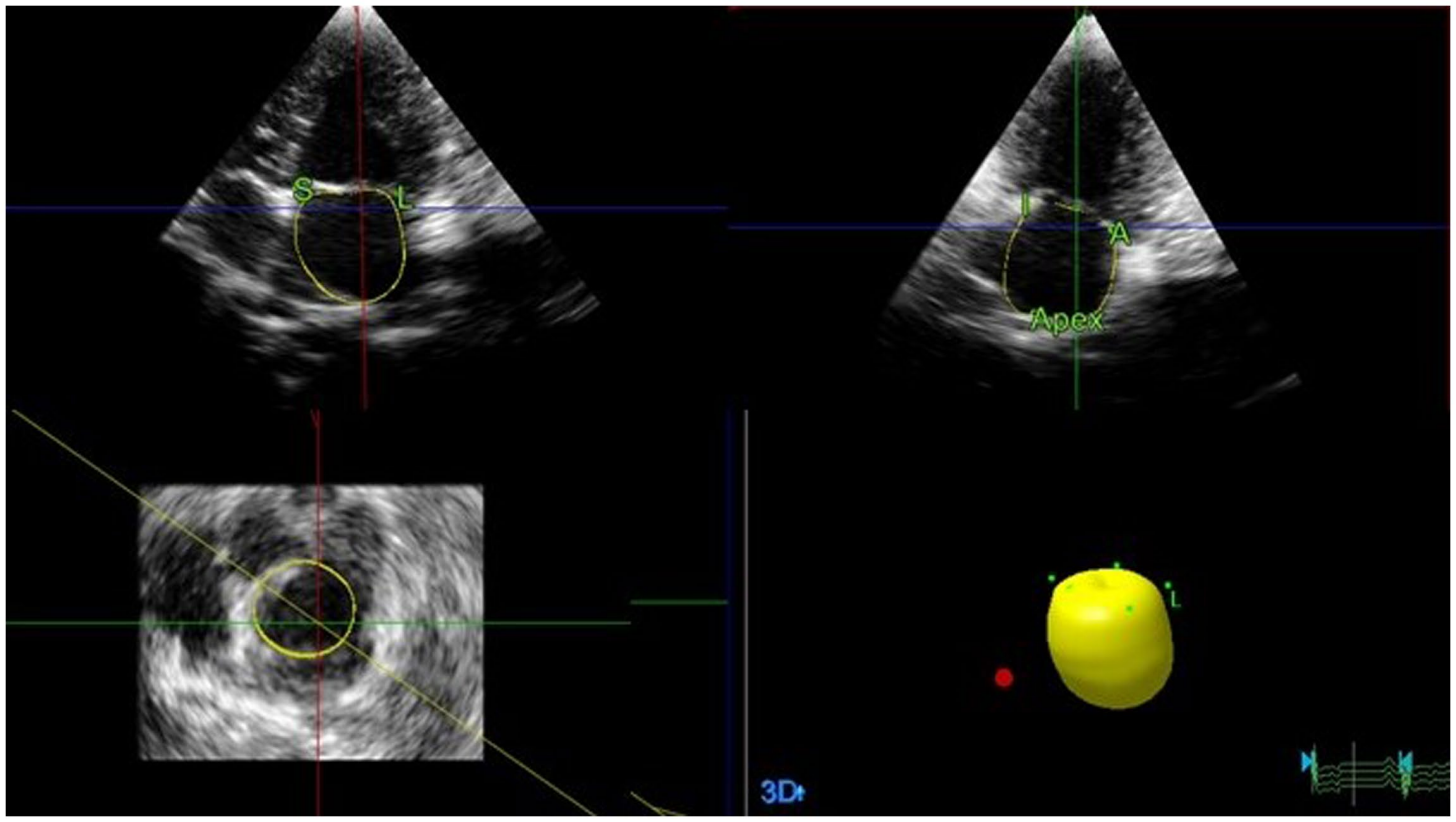
Representative image of LAV obtained using RT3DE. To analyze LAVs obtained from RT3DE images, pyramidal data were displayed in three different planes (lateral, sagittal, and coronal). These were then adjusted by manually shifting the horizontal and vertical lines. Once the image was properly aligned to outline the endocardial border, five anatomical reference points and a semi-automatically generated endocardial cast were manually identified. Among these points, two were placed on the septal and lateral mitral valve annuli, whereas the remaining two were placed on the orthogonal image in anterior and posterior orientations. The final reference point was placed on the dorsal border of the left atrium. Subsequently, the software automatically generated an LA endocardial cast using a deformable shell model. The endocardial border was manually adjusted during all examinations, whenever necessary. LA, Left atrial; LAV, Left atrial volume; and RT3DE, Real-time three-dimensional echocardiography.

**Figure 5:**
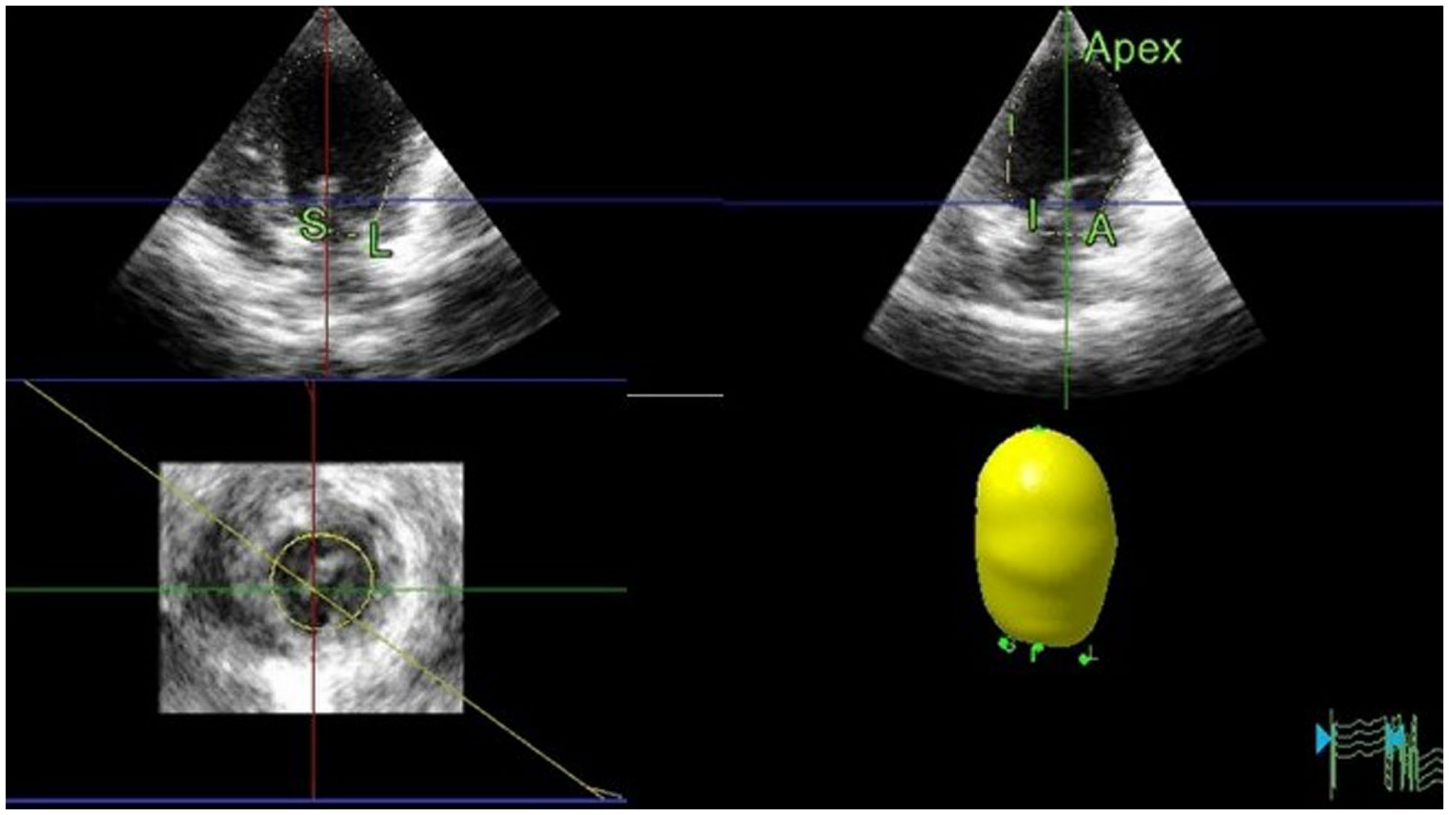
Representative image of LV volume obtained using RT3DE. To assess the LV volumes and ejection fraction using RT3DE, four reference points were manually defined at the endocardial border of the mitral annulus hinge points in both the four-chamber and two-chamber views. A fifth reference point was defined at the endocardial border of the apex in either view in end-diastole and end-systole. An automated detection process was used to trace the endocardial border and create a cast of the LV cavity. Once the volume was computed, endocardial border detection was verified for accuracy and manually edited as required, including the endocardial borders and papillary muscles. LV, Left ventricular; RT3DE, Real-time three-dimensional echocardiography.

Offline analyses were performed by the same investigator (I-SW) using the system software (see text footnote 2). The investigator was blinded to the dogs’ statuses and 2D echocardiographic analysis. The LAV (Figure 4) and LV volume (Figure 5) were obtained using RT3DE, as previously described (15, 20). Maximum and minimum LAV assessment was performed using the abovementioned methods. All LAV and LV volumes obtained using RT3DE were indexed to BW (14, 18). The volumes and EF were calculated automatically using the same system software (see text footnote 2).

### 2.5 Intra-observer measurement variability

One month after completing the initial phase of the study, a reproducibility study was performed to test intra-observer measurement variability. The same observer who initially analyzed the selected images remeasured 10 randomly selected dogs, analyzing the same beat twice (blinded from the previous measurements). Next, the coefficient of variation (CV), expressed as a percentage, was calculated using the following formula: CV = (mean difference between measurement/mean of measurements)/100. The extent of variability was arbitrarily defined as very low variability (< 5%), low variability (5–15%), moderate variability (16–25%), or high variability (>25%).

### 2.6 Statistical analysis

Data were analyzed using commercial software^3^^,^^4^ and presented as medians (25 and 75th percentiles). Normality was assessed using the Shapiro–Wilk test. Continuous data were analyzed using the Mann–Whitney *U* test. Categorical indices were compared using the chi-squared or Fisher’s exact tests, and correlations were assessed using Spearman’s coefficients.

Logistic regression analysis was used to assess the factors associated with post-capillary PH. The collinearity of variables was assessed using the variance inflation factor (> 10 was considered excessive). Next, significant associations from the univariable analysis (*p* < 0.2) were analyzed using a multivariable logistic regression model, with backward stepwise selection depending on likelihood ratio statistics.

Receiver operating characteristic (ROC) curves were used to evaluate the diagnostic efficacy of the independent parameters to predict post-capillary PH. The cutoff points derived from the ROC curves (using the Youden index) were used to assess the predictive ability of LAV obtained using 2DE and RT3DE for post-capillary PH. Agreement between 2DE and RT3DE LAVs was analyzed using the Bland–Altman method (21). Statistical significance was set at *p* < 0.05.

## 3 Results

### 3.1 Characteristics of the study population at admission

A total of 49 dogs (35 without PH and 14 with PH) were enrolled in this study, comprising the following breeds: Maltese (16), Poodle (10), Pomeranian (6), Shih Tzu (4), Chihuahua (3), mixed breed (2), Schnauzer (2), Spitz (2), Miniature Pinscher (2), Toy Poodle (1), and Cocker Spaniel (1). Clinical characteristics, treatments received at the time of echocardiography, and selected radiographic indices associated with cardiomegaly are summarized in Table 1.

**Table 1:** Characteristics of the study population at admission.

Variables	Dogs with MMVD without PH (*n* = 35)	Dogs with MMVD and PH (*n* = 14)	*p* value
Age (years)	11.0 (8.0–13.0)	10.0 (8.0–11.3)	0.270
Sex (male/female)	21/14	9/5	0.523
Body weight (kg)	4.0 (3.0–5.8)	3.8 (3.2–5.8)	0.642
SBP (mmHg)	144 (130–148)	135 (121–141)	0.030^*^
Heart rate (beats/min)	138 (126–162)	146 (129–183)	0.381
VHS	11.1 (10.7–11.4)	12.2 (11.1–13.2)	0.007^*^
VLAS	2.8 (2.5–3.1)	3.0 (2.8–3.1)	0.477
ACVIM stage (B2/C/D)	19/16/0	7/5/2	0.072
Medications (*n*, %)			
	ACEi (*n* = 19, 54.3%)	ACEi (*n* = 11, 78.6%)	
	Diuretics (*n* = 23, 65.7%)	Diuretics (*n* = 9, 64.3%)	
	Pimobendan (*n* = 22, 62.8%)	Pimobendan (*n* = 9, 64.3%)	
	Spironolactone (*n* = 19, 54.3%)	Spironolactone (*n* = 9, 64.3%)	
	CCB (*n* = 7, 20.0%)	CCB (*n* = 2, 14.3%)	
	PDE5i (*n* = 0, 0%)	PDE5i (*n* = 6, 42.9%)	

Continuous data are presented as medians and interquartile ranges (^*^p < 0.05). ACEi, Angiotensin-converting enzyme inhibitors; ACVIM, American College of Veterinary Internal Medicine; CCB, Calcium channel blocker; MMVD, Myxomatous mitral valve disease; PDE5i, Phosphodiesterase-5 inhibitor; PH, Pulmonary hypertension; SBP, Systolic blood pressure; VHS, Vertebral heart size; and VLAS, Vertebral left atrial score.

### 3.2 Two-dimensional and real-time three-dimensional echocardiography

Baseline 2DE and RT3DE variables are presented in Tables 2, 3. Dogs with MMVD were classified by the presence of PH. Dogs with MMVD and PH had significantly higher values in the following: 2DE maximum LAV indexed to BW (LAVi max), 2DE minimum LAV indexed to BW (LAVi min), 2DE LA EF, 2DE LV end-diastolic volume (EDV) indexed to body weight (EDVi), 2DE LV end-systolic volume (ESV) indexed to BW (ESVi), RT3DE LAVi max, RT3DE LAVi min, RT3DE LA EF, RT3DE LV EDVi, and RT3DE LV ESVi. However, 2DE LV EF and RT3DE were not significantly different between dogs with and without PH.

**Table 2:** Two-dimensional echocardiography-derived variables of the study population.

Variables	Dogs with MMVD without PH (*n* = 35)	Dogs with MMVD and PH (*n* = 14)	*p* value
LVIDDn	1.79 (1.49–2.01)	2.00 (1.76–2.20)	0.033^*^
LA/Ao	1.72 (1.65–1.89)	2.28 (1.81–2.44)	0.010^*^
MR velocity (m/s)	5.80 (5.53–6.22)	5.27 (4.55–5.70)	0.016^*^
TR velocity (m/s)^‡^	1.9 (2.1–2.4)	3.3 (3.0–4.0)	< 0.001^**^
AT (ms)	75.5 (62.0–90.5)	65.0 (55.8–77.0)	0.041^*^
AT/ET	0.4 (0.4–0.5)	0.4 (0.3–0.4)	0.215
MPA/Ao	0.9 (0.8–1.0)	1.1 (1.0–1.2)	0.020^*^
E peak velocity (cm/s)	103.1 (72.6–121.8)	126.7 (106.3–156.4)	0.006^*^
A peak velocity (cm/s)	95.0 (78.7–107.9)	83.3 (44.7–117.1)	0.252
E/A	1.0 (0.8–1.2)	2.0 (0.9–2.8)	0.013^*^
E′/A′	0.8 (0.6–1.1)	1.6 (0.7–2.0)	0.007^*^
E/E′	12.4 (9.8–17.4)	13.7 (8.9–15.5)	0.642
IVRT	60 (51–65)	51 (48–71)	0.393
E/IVRT	1.6 (1.3–2.3)	2.4 (2.1–2.9)	0.005^*^
2DE LAV max (mL)	6.8 (4.4–8.3)	8.6 (7.1–24.9)	0.023^*^
2DE LAVi max (mL/kg)	1.4 (1.1–2.0)	2.8 (1.8–3.4)	0.002^*^
2DE LAV min (mL)	2.6 (1.5–3.5)	4.3 (2.1–15.9)	0.028^*^
2DE LAVi min (mL/kg)	0.5 (0.4–1.0)	1.3 (0.7–2.3)	0.003^*^
2DE LA EF (%)	63.8 (54.8–63.8)	48.6 (35.4–64.2)	0.024^*^
2DE EDV (mL)	12.8 (10.5–16.9)	18.3 (9.7–29.6)	0.219
2DE EDVi (mL/kg)	2.9 (2.3–4.1)	4.4 (3.2–5.6)	0.019^*^
2DE ESV (mL)	5.5 (4.2–6.5)	6.6 (4.0–11.2)	0.288
2DE ESVi (mL/kg)	1.2 (1.0–1.7)	1.8 (1.3–2.1)	0.022^*^
2DE LV EF (%)	56.2 (53.0–62.8)	58.9 (56.1–64.5)	0.144

Continuous data are presented as medians and interquartile ranges (^*^p < 0.05, ^**^p < 0.001). 2DE, Two-dimensional echocardiography; AT, Acceleration time of peak pulmonary artery flow; AT/ET, Ratio of acceleration time of peak pulmonary artery flow to ejection time of pulmonary artery flow; E/A, Ratio of transmitral flow E wave velocity to A wave velocity; E′/A′, Peak velocity of early diastolic mitral annular motion to peak velocity of diastolic mitral annular motion as determined by pulsed-wave Doppler; EDV, End-diastolic volume; EDVi, End-diastolic volume indexed to body weight; E/E′, Transmitral flow E wave velocity to peak velocity of early diastolic mitral annular motion; EF, Ejection fraction; E/IVRT, Ratio of transmitral flow E wave velocity to isovolumic relaxation time; ESV, End-systolic volume; ESVi, End-systolic volume indexed to body weight; IVRT, Isovolumic relaxation time; LA, Left atrium; LA/Ao, Ratio of the left atrial diameter to the aortic diameter; LAV max, Maximum left atrial volume; LAVi max, Maximum left atrial volume indexed to body weight; LAV min, Minimum left atrial volume; LAVi min, Minimum left atrial volume indexed to body weight; LV, Left ventricle; LVIDDn, Left ventricular end-diastolic internal diameter normalized for body weight; MMVD, Myxomatous mitral valve disease; MPA/Ao, Ratio of main pulmonary artery to aortic diameter; MR, Mitral valve regurgitation; PH, Pulmonary hypertension; TR, Tricuspid regurgitation. ^‡^TR velocity was measured in seven dogs with MMVD without PH.

**Table 3:** RT3DE-derived variables of the study population.

Variables	Dogs with MMVD without PH (*n* = 35)	Dogs with MMVD and PH (*n* = 14)	*p* value
RT3DE LAV max (mL)	6.9 (4.5–8.6)	8.4 (7.5–24.6)	0.016^*^
RT3DE LAVi max (mL/kg)	1.4 (1.2–2.1)	2.9 (2.0–3.7)	< 0.001^**^
RT3DE LAV min (mL)	2.5 (1.5–3.6)	4.2 (3.1–16.0)	0.002^*^
RT3DE LAVi min (mL/kg)	0.5 (0.4–1.0)	1.3 (1.0–2.5)	< 0.001^**^
RT3DE LA EF (%)	63.0 (56.1–68.1)	43.8 (33.0–52.8)	< 0.001^*^
RT3DE EDV (mL)	12.9 (8.8–17.2)	18.1 (9.2–28.8)	0.160
RT3DE EDVi (mL/kg)	2.9 (2.3–3.9)	4.4 (3.5–5.3)	0.013^*^
RT3DE ESV (mL)	5.2 (4.1–6.5)	7.0 (3.6–9.5)	0.180
RT3DE ESVi (mL/kg)	1.3 (0.9–1.5)	1.7 (1.4–2.1)	0.005^*^
RT3DE LV EF (%)	57.1 (51.4–64.0)	62.0 (54.2–66.0)	0.400

Continuous data are presented as medians and interquartile ranges (^*^p < 0.05, ^**^p < 0.001). EDV, End-diastolic volume; EDVi, End-diastolic volume indexed to body weight; EF, Ejection fraction; ESV, End-systolic volume; ESVi, End-systolic volume indexed to body weight; LA, Left atrium; LAV, Left atrial volume; LAV max, Maximum left atrial volume; LAVi max, Maximum left atrial volume indexed to body weight; LAV min, Minimum left atrial volume; LAVi min, Minimum left atrial volume indexed to body weight; LV, Left ventricle; MMVD, Myxomatous mitral valve disease; PH, Pulmonary hypertension; and RT3DE, Real-time three-dimensional echocardiography.

### 3.3 Predictive value of 2DE- and RT3DE-derived variables on PH in dogs with MMVD by logistic regression analysis

Logistic regression analysis was used to determine the parameters that can be used to predict post-capillary PH. Table 4 shows the results of univariable logistic regression analysis for evaluating the predictive value of 2DE- and RT3DE-derived variables for PH. After adjusting for confounding factors, the input parameters for the multivariable logistic regression model were as follows (VIF = 8.28): LA/Ao, main pulmonary artery to aortic diameter ratio (MPA/Ao), transmitral flow E wave velocity to isovolumic relaxation time ratio (E/IVRT), LVIDDn, transmitral flow E wave velocity to A wave velocity ratio (E/A), RT3DE LA EF (%), RT3DE LAVi min, and RT3DE LV ESVi. The RT3DE LAVi min was the only significant independent predictor of post-capillary PH after multivariable analysis (*p* = 0.003) (Table 5).

**Table 4:** Univariable analysis for the predictive value of 2DE and RT3DE for PH in dogs with MMVD.

	Univariable analysis	
Variables	OR (95% CI)	*p* value
LA/Ao^†^	1.28 (1.07–1.53)	0.007^*^
MPA/Ao^†^	1.64 (1.07–2.51)	0.024^*^
E/IVRT^†^	1.16 (1.04–1.30)	0.008^*^
LVIDDn^†^	1.30 (1.03–1.63)	0.025^*^
E/A^†^	1.16 (1.05–1.28)	0.004^*^
*2DE volume*		
2DE LA EF (%)	0.95 (0.90–0.99)	0.020^*^
2DE LV EF (%)	1.05 (0.96–1.15)	0.263
2DE LAVi max (mL/kg)	3.38 (1.51–7.57)	0.003^*^
2DE LAVi min (mL/kg)	7.81 (1.86–32.92)	0.005^*^
2DE LV EDVi (mL/kg)	1.80 (1.10–2.95)	0.020^*^
2DE LV ESVi (mL/kg)	4.40 (1.15–16.91)	0.031^*^
*RT3DE volume*		
RT3DE LA EF (%)	0.89 (0.84–0.95)	0.001^*^
RT3DE LV EF (%)	1.04 (0.96–1.12)	0.341
RT3DE LAVi max (mL/kg)	3.27 (1.53–6.96)	0.002^*^
RT3DE LAVi min (mL/kg)	12.87 (2.40–68.99)	0.003^*^
RT3DE LV EDVi (mL/kg)	1.97 (1.13–3.42)	0.017^*^
RT3DE LV ESVi (mL/kg)	9.88 (1.72–56.71)	0.010^*^

2DE, Two-dimensional echocardiography; CI, Confidence interval; E/A, Ratio of transmitral flow E wave velocity to A wave velocity; EDVi, End-diastolic volume indexed to body weight; EF, Ejection fraction; E/IVRT, Ratio of transmitral flow E wave velocity to isovolumic relaxation time; ESVi, End-systolic volume indexed to bodyweight; LA, Left atrium; LA/Ao, Ratio of left atrial to aortic root; LAVi max, Maximum left atrial volume indexed to body weight; LAVi min, Minimum left atrial volume indexed to body weight; LV, Left ventricle; LVIDDn, Left ventricular end-diastolic internal diameter normalized for body weight; MMVD, Myxomatous mitral valve disease; MPA/Ao, Ratio of main pulmonary artery to aortic diameter; OR, Odds ratio; PH, Pulmonary hypertension; RT3DE, Real-time three-dimensional echocardiography; ^*^p < 0.05, ^†^per 0.1 increase.

**Table 5:** Final multivariable analysis model to identify independent PH predictors in dogs with MMVD.

	Multivariable analysis	
Variable	OR (95% CI)	*p* value
RT3DE LAVi min (mL/kg)	12.86 (2.40–68.99)	0.003^*^

CI, Confidence interval; LAVi min, Minimum left atrial volume indexed to body weight; MMVD, Myxomatous mitral valve disease; OR, Odds ratio; PH, Pulmonary hypertension; RT3DE, Real-time three-dimensional echocardiography. ^*^p < 0.05.

### 3.4 Diagnostic accuracy of 2DE- and RT3DE-derived variables for PH occurrence in dogs with MMVD

**Figure 6:**
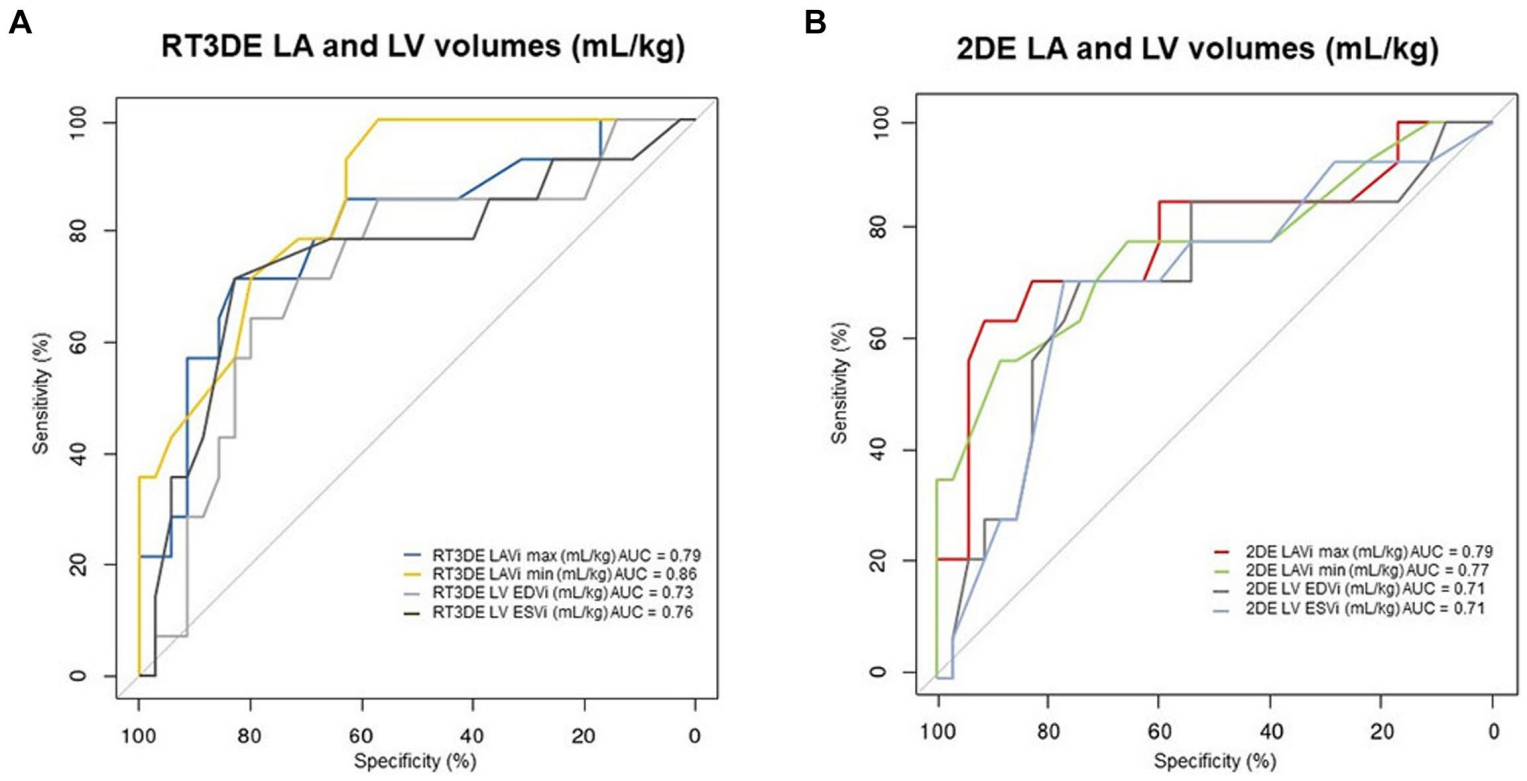
ROC curves for detecting PH in dogs with MMVD. ROC curves represent the predictive value of the LA volume measurement using RT3DE **(A)** and 2DE **(B)**, with their corresponding AUC values. 2DE, Two-dimensional echocardiography; AUC, Area under the curve; LA, Left atrium; LAVi max, Maximum LA volume indexed to body weight; LAVi min, Minimum LA volume indexed to body weight; LV, Left ventricle; LV EDVi, Left ventricular end-diastolic volume indexed to body weight; LV ESVi, Left ventricular end-systolic volume indexed to body weight; MMVD, Myxomatous mitral valve disease; PH, Pulmonary hypertension; ROC, Receiver operating characteristic; and RT3DE, Real-time three-dimensional echocardiography.

**Figure 7:**
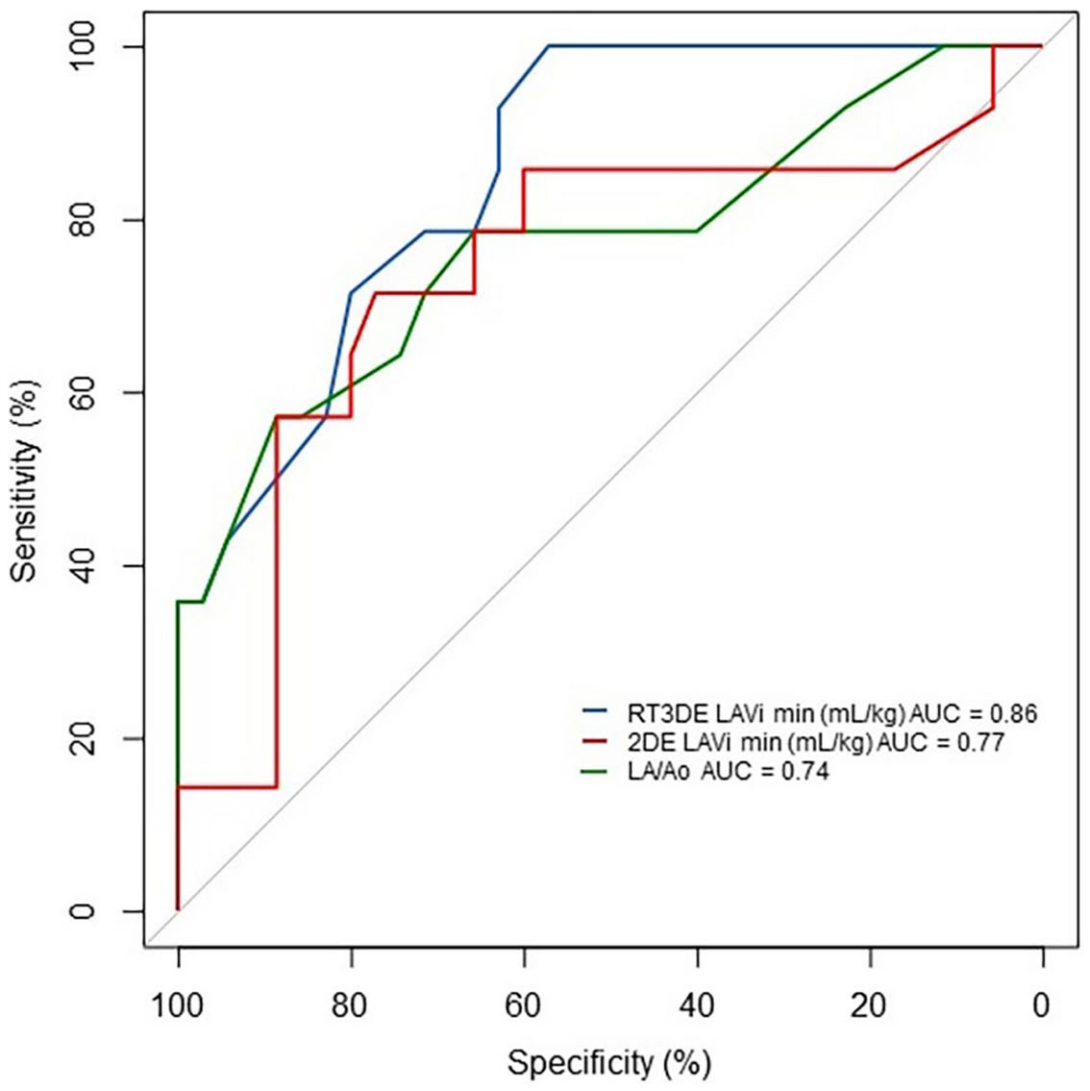
ROC curve of LAVi min and LA/Ao for detecting PH in dogs with MMVD. RT3DE LAVi min showed a higher diagnostic value for predicting the presence of post-capillary PH than that of LA/Ao. 2DE, Two-dimensional echocardiography AUC, Area under the curve; LA/Ao, Left atrial to aortic root ratio; LAVi min, Minimum left atrial volume indexed to body weight; MMVD, Myxomatous mitral valve disease; PH, Pulmonary hypertension; ROC, Receiver operating characteristic; and RT3DE, Real-time three-dimensional echocardiography.

The results of the ROC analysis and cutoff values for predicting post-capillary PH are summarized in Table 6 and Figure 6. The RT3DE LAVi min showed the best diagnostic accuracy for identifying PH, with the highest AUC value (0.86, *p* < 0.001). Two cutoff values were determined from the ROC analysis, one for optimal test efficiency (determined by the highest Youden index) and one for maximal specificity (least number of false positives). The optimal cutoff of RT3DE LAVi min for detecting PH was >0.55 mL/kg. Cutoffs for maximum specificity (fewest false positives) for RT3DE LAVi min was >2.0 mL/kg. According to the ROC analysis, RT3DE LAVi min showed a higher diagnostic value for predicting the presence of post-capillary PH than LA/Ao (Figure 7).

**Table 6:** Diagnostic accuracy of LAVi using 2DE and RT3DE for detecting PH in dogs with MMVD.

Variables	AUC (95% CI)	Cutoff	Sensitivity	Specificity	Youden index	*p* value
*2DE*						
2DE LAVi max (mL/kg)	0.79 (0.64–0.95)	**> 2.55**	**0.64**	**0.91**	**0.55**	0.002^*^
		> 4.00	0.21	1.00	0.21	
2DE LAVi min (mL/kg)	0.77 (0.61–0.93)	**> 1.15**	**0.57**	**0.89**	**0.46**	0.003^*^
		> 1.65	0.36	1.00	0.36	
*RT3DE*						
RT3DE LAVi max (mL/kg)	0.80 (0.65–0.94)	**> 2.25**	**0.71**	**0.83**	**0.54**	0.001^*^
		> 4.10	0.21	1.00	0.21	
RT3DE LAVi min (mL/kg)	0.86 (0.75–0.96)	**> 0.55**	**1.00**	**0.57**	**0.57**	< 0.001^**^
		> 2.00	0.36	1.00	0.36	

2DE, Two-dimensional echocardiography; AUC, Area under the curve; CI, Confidence interval; LAVi, Left atrial volume indexed to body weight; LAVi max, Maximum left atrial volume indexed to body weight; LAVi min, Minimum left atrial volume indexed to body weight; MMVD, Myxomatous mitral valve disease; PH, Pulmonary hypertension; and RT3DE, Real-time three-dimensional echocardiography. The bold cutoff value indicates the highest Youden index. ^*^p < 0.05, ^**^p < 0.001.

### 3.5 Agreement between 2DE and RT3DE

**Figure 8:**
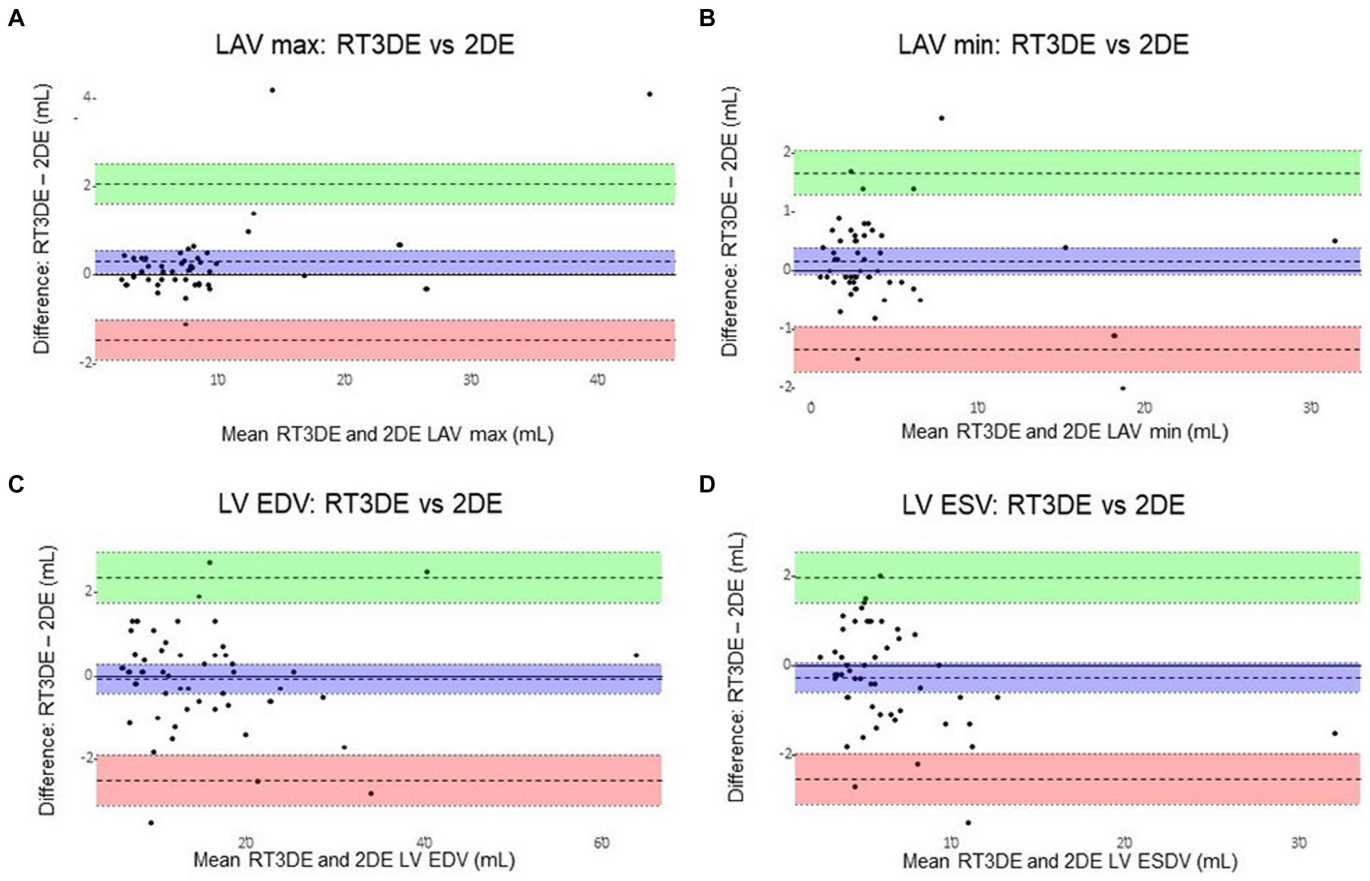
Bland–Altman plots of LA and LV volumes obtained using 2DE and RT3DE. The difference between 2DE and RT3DE measurements was plotted against the mean value of the two measurements. The mean difference (mean bias) and standard deviation(s) of the differences were calculated. Solid lines represent the mean differences (bias) and dashed lines represent the 95% limits of agreement (±1.96 standard deviations from the mean between the two techniques). 2DE slightly underestimated the maximum LAV compared with RT3DE (bias = 0.30 mL; 95% limit of agreement: −1.46 to 2.06) **(A)**. Similarly, 2DE underestimated the minimum LAV (bias = 0.16 mL; 95% limit of agreement: −1.34 to 1.66) **(B)**. 2DE slightly overestimated the LV EDV to RT3DE (bias = –0.07 mL; 95% LoA: –2.49 to 2.34) **(C)**. And 2DE overestimated the LV ESV compared to RT3DE (bias = –0.28 mL; 95% LoA: –2.53 to 1.95) **(D)**. 2DE, Two-dimensional echocardiography; LA, Left atrial; LAV, Left atrial volume; LAV max, Maximum left atrial volume; LAV min, Minimum left atrial volume; LV, Left ventricular; LV EDV, Left ventricular end-diastolic volume; LV ESV, Left ventricular end-systolic volume; and RT3DE, Real-time three-dimensional echocardiography.

The Bland–Altman analysis results of the volume parameters measured using 2DE compared to those measured using RT3DE are summarized in Table 7 and Figure 8. 2DE slightly underestimated the maximum LAV compared to RT3DE [bias = 0.30 mL; 95% limit of agreement (LoA): −1.46 to 2.06]. Similarly, 2DE underestimated the minimum LAV (bias = 0.16 mL; 95% LoA: −1.34 to 1.66).

**Table 7:** Bias and limits of agreement between 2DE and RT3DE for LA and LV volume measurements.

Variables	Bias (mL)	Limits of agreement
Maximum LAV	0.30	−1.46 to 2.06
Minimum LAV	0.16	−1.34 to 1.66
LV EDV	−0.07	−2.49 to 2.34
LV ESV	−0.28	−2.53 to 1.95

2DE, Two-dimensional echocardiography; EDV, End-diastolic volume; ESV, End-systolic volume; LA, Left atrial; LAV, Left atrial volume; LV, Left ventricular; and RT3DE, Real-time three-dimensional echocardiography.

### 3.6 Reproducibility

All volumes measured using 2DE and RT3DE, with CV values always below 16%, showed good reproducibility. The CVs for intra-observer variability were 2.6% for 2DE LAV max, 4.1% for 2DE LAVi min, 2.6% for LV EDV, 5.6% for 2DE LV ESV, 3.1% for RT3DE LAV max, 5.8% for RT3DE LAV min, 3.0% for RT3DE LV EDV, and 2.9% for LV ESV.

## 4 Discussion

Generally, 2DE and RT3DE are performed on dogs and humans to noninvasively assess LAV associated with chronic LV diastolic dysfunction. In humans, LAV obtained using RT3DE showed great predictive value for post-capillary PH (13). However, no studies have investigated LAV obtained using RT3DE as a predictor of post-capillary PH in dogs with MMVD. Therefore, the present study evaluated the predictive value of LAV obtained using RT3DE for post-capillary PH in dogs with MMVD. To the best of our knowledge, this is the first study to evaluate the clinical relevance of LAV obtained using RT3DE in dogs with MMVD, stratified by the presence of PH.

Indexing measured volumes to body size is essential for accurate comparisons. In humans, this commonly involves indexing LAV and LV volume to BSA (9, 12, 22). A large-scale study showed that volumetric measurement using 2DE SMOD showed the best correlation with BW (11, 19); however, this remains controversial in veterinary medicine (23, 24). Furthermore, previous studies have reported that LA and LV volumes indexed to BW using 2DE and RT3DE showed great predictive and prognostic value in dogs with MMVD (15, 25, 26). Therefore, our study reports the LAV and LV volume indexed to BW.

Maximum LAV reflects the severity and chronicity of LV diastolic dysfunction (12). The maximum LAV indexed to the BSA is a well-known independent predictor of human cardiovascular outcomes (22). Similarly, the maximum LAV indexed to BW showed significant predictive and prognostic values in dogs with MMVD (15, 26).

In the present study, both 2DE and RT3DE LAVi max were significantly higher in dogs with MMVD and PH than in those without PH (*p* = 0.002, *p* < 0.001, respectively). Furthermore, the univariable analysis showed that 2DE and RT3DE LAVi max were significant predictors of post-capillary PH (*p* = 0.003, *p* = 0.002, respectively). The ROC analyses revealed that both 2DE and RT3DE LAVi max had significant power to predict the presence of PH in dogs with MMVD (*p* = 0.002, *p* = 0.001, respectively). Therefore, LAVi max obtained using 2DE and RT3DE can be a useful predictor of post-capillary PH in dogs with MMVD.

Recently, studies have shown that the minimum LAV better reflects LV end-diastolic pressure than the maximum LAV in humans (6, 27) owing to the continuous exposure of the LA to LV pressure during diastole (6). Moreover, the minimum LAV increased more prominently than the maximum LAV with worsening diastolic function, increasing even in cases of mild diastolic dysfunction, whereas the maximum LAV increased in later stages of diastolic dysfunction (28). Accordingly, the minimum LAV is considered a better predictor of cardiac events (e.g., atrial fibrillation, heart failure, and death) than the maximum LAV in humans (13, 29). Conversely, a previous study revealed no additional value in measuring the LAVi min in dogs with MMVD (30).

In the present study, both 2DE and RT3DE LAVi min were significantly higher in dogs with MMVD and PH than in those without PH (*p* = 0.003, *p* < 0.001, respectively). Univariable analysis revealed that both LA and LV volumes obtained using 2DE and RT3DE indexed to BW and 2DE-derived variables were significantly associated with the presence of PH (*p* = 0.005, *p* = 0.003, respectively). A multivariable logistic regression analysis was performed to further investigate independent predictors of post-capillary PH. On adjusting for multicollinearity, correlations between variables, and clinical relevance, our results revealed that RT3DE LAVi min was the only significant predictor of post-capillary PH (*p* = 0.003).

Receiver operating characteristic analysis was performed to evaluate the diagnostic value of RT3DE LAV min. The RT3DE LAVi min showed the best diagnostic accuracy for identifying PH, with the highest AUC value (0.86, *p* < 0.001), in line with previous studies reporting that the minimum LAV indexed to BSA is an independent determinant of post-capillary PH in humans (13, 31). The optimal cutoff of RT3DE LAVi min for detecting PH in dogs with MMVD was >0.55 mL/kg (high sensitivity but a relatively low specificity of 0.57). Cutoffs for maximum specificity (fewest false positives) for RT3DE LAVi min was >2.0 mL/kg, which was associated with the management of PH (e.g., prescription of PDE5i or predicting prognosis). These results may provide additional information for diagnosis and stratification of PH in dogs with MMVD.

The univariable analysis demonstrated that the LA/Ao, MPA/Ao, E/IVRT, LVIDDn, and E/A were significantly associated with PH; however, these associations were not significant in the multivariable analysis. As an accurate volume-dependent parameter, RT3DE LAVi min could reflect increased LA preload or LV diastolic dysfunction better than LA/Ao, E/IVRT, LVIDDn, and E/A. Therefore, RT3DE LAVi min was superior to the other 2DE-derived variables and was considered the best predictor of post-capillary PH in this study. However, additional potential confounding variables such as indices of right heart volume and function were not evaluated. Further research including simultaneous analysis of volumes and functions of the four cardiac chambers is warranted to comprehensively understand the parameters associated with PH in dogs with MMVD.

LA EF exhibited a negative correlation with both LV systolic and diastolic dysfunction in human patients (32). Progressive reduction in LA EF was observed concomitantly with worsening diastolic dysfunction (28). In the previous study, the median LA EF of ≤15 kg healthy dogs obtained using RT3DE was 61.5% (18). In this study, the median LA EF of dogs with MMVD and PH was 43.8%. LA EF obtained using 2DE and RT3DE were significantly lower in dogs with MMVD and PH than in those without PH (*p* = 0.024, *p* < 0.001, respectively). Furthermore, LA EF obtained using 2DE and RT3DE was univariately associated with post-capillary PH. The reduction in LA EF may be attributed to the concomitant development of post-capillary PH.

We assessed the LV volume owing to LA and LV interdependency. In the present study, BW-indexed LV volumes obtained using both 2DE and RT3DE were significantly higher in dogs with MMVD and PH than in those without PH. Furthermore, univariable analysis revealed that LV volumes obtained using both 2DE and RT3DE were significantly associated with PH. Multivariable analysis revealed that RT3DE ESVi was not an independent predictor of PH, in line with the results of a previous study showing that LV ESV was not an independent predictor of post-capillary PH (31, 33). Left ventricular volumes may be confounded by the effects of changes in LA loading conditions.

The ROC analysis revealed that LA dilatation had a higher diagnostic accuracy than LV enlargement (Figure 6). Elevated pressures in the right ventricle, constituting a part of the pathophysiology of post-capillary PH, can cause flattening of the interventricular septum (34). Consequently, reduced EDV and ESV have been observed with preserved global systolic function assessed using the EF (35). Furthermore, LA enlargement precedes LV enlargement in cardiac remodeling owing to pressure overload since the left atrium adapts to elevated LA pressure before significant LV changes occur. In dogs with MMVD, significant LV EDV and ESV enlargement assessed by RT3DE were found only in severely affected dogs (36). These results confirmed the importance of early detection of LA enlargement in dogs with MMVD. Moreover, previous studies showed that the LA/Ao can underestimate the LAV when comparing LAV obtained using 2DE and RT3DE (37, 38). In the ROC analysis, RT3DE LAVi min showed a higher diagnostic value for predicting the presence of PH compared with LA/Ao, which is the most commonly used variable to assess LA enlargement in veterinary medicine (Figure 7). Therefore, RT3DE LAVi min may provide additional diagnostic value to predict post-capillary PH in dogs with MMVD.

Tricuspid regurgitation velocity is considered to be the most reliable tool to predict the presence of PH; however, this measurement depends on multiple factors, such as poor patient cooperation, labored respiration, right ventricular function, pulmonary artery pressure, and pulmonary vascular resistance (4). Clinicians should consider the limitations of echocardiography and the inaccuracy, variability, and imprecision that can be potentially encountered when using echocardiography to estimate pulmonary artery pressure in individual dogs. When post-capillary PH is suspected but the measured peak TR velocity is not adequately high, the RT3DE LAVi min may be useful for predicting the presence of post-capillary PH in dogs with MMVD.

As expected, the Bland–Altman analyses revealed an underestimation of LAVi max (bias = 0.30 mL; LoA: −1.46 to 2.06) and LAVi min (bias = 0.16 mL; 95% LoA: −1.34 to 1.66) using 2DE compared to RT3DE in this study. In line with previous studies, the results of the present study confirmed that 2DE systemically underestimates LAV more than RT3DE due to its geometric assumptions and foreshortening (8). In addition, a previous study demonstrated that manual border tracing errors may contribute more to the underestimation of LAV in 2DE compared to RT3DE (6). Considering the median LAVs and the bias between LAVi max and min, these potential underestimations could have affected the minimum LAV more than the maximum LAV in this study.

The RT3DE is feasible and repeatable for evaluating LAV in humans (9, 39, 40) and dogs (23, 38, 41). In the present study, intraobserver variability was clinically acceptable (CV < 16%) with good reproducibility (2.6–5.8%) for all LAV and LV volume measurements. Similar data have been reported in dogs, with intraobserver variability coefficients of measurements ranging between 3.6 and 5.8% for LAV obtained using 2DE SMOD and RT3DE from left apical four-chamber view (19). However other studies reported intraobserver variability coefficients ranging from 4.7 to 15.9% (23, 42). Reduced accuracy at larger LAV may be attributed to the views used for image derivation. Further, the effect size would be greater with larger LA, which have longer perimeters (8). In this study, the study population comprised mostly small-sized dogs; thus the reproducibility might have been relatively low compared to that reported in previous studies.

This study had some limitations. First, this was a retrospective study that only included dogs with a sinus rhythm and good image quality, potentially introducing selection bias. Second, this single-center study involved only one examiner, limiting the generalizability of the results and introducing observer bias. Third, the potential influence of medications known to affect the cardiovascular system was not thoroughly explored. Several drug combinations were used; therefore, analyzing their therapeutic effects on PH was not possible. Many of these medications could have relevant hemodynamic effects on LAV. Finally, although intravariability in the present study was considered to be good to acceptable, intraobserver variability was not analyzed.

In conclusion, LAVi obtained using 2DE and RT3DE is a useful predictor of post-capillary PH in dogs with MMVD. In particular, the RT3DE LAVi min showed the best predictive value for detecting PH. The superior diagnostic accuracy of RT3DE LAVi min highlights its potential clinical use for prompt and precise diagnosis and management of post-capillary PH in dogs with MMVD. Further large-scale multicenter studies evaluating the prognostic and predictive value of LAVi obtained using RT3DE in dogs with MMVD and PH are warranted to establish its validity, generalizability, and applicability in various clinical settings.

## Data availability statement

The datasets presented in this study can be found in online repositories. The names of the repository/repositories and accession number(s) can be found at: https://doi.org/10.6084/m9.figshare.26426734.v1.

## Ethics statement

The animal studies were approved by Institutional Animal Care and Use Committee of Konkuk University. The studies were conducted in accordance with the local legislation and institutional requirements. Written informed consent was not obtained from the owners for the participation of their animals in this study because retrospective nature of this study.

## Author contributions

I-SW: Conceptualization, Data curation, Formal analysis, Investigation, Methodology, Software, Validation, Visualization, Writing – original draft, Writing – review & editing. J-HK: Conceptualization, Investigation, Methodology, Software, Supervision, Validation, Writing – original draft, Writing – review & editing.

## Footnotes

^1^EPIQ 7, Philips Ultrasound, Bothell, WA, United States

^2^QLAB advanced quantification, version 13.0, Philips Ultrasound, Bothell, WA, United States

^3^SPSS statistical package version 26.0, IBM, Armonk, NY, United States

^4^R, version 4.04, R Development Core Team, Vienna, Austria.
